# Further Account of Miss M'Evoy

**Published:** 1817-11

**Authors:** T. Glover


*For the London Medical and Physical Journal.*

*Further Account of Miss M'Evoy* [see page 291]. Extracted from the Annals of Philosophy.

"*Extraordinary Case of a Blind Young Person who can read by the Points of her Fingers;* by the Rev. T. GLOVER.

(To Dr. Thomson.)

SIR,

BEING lately on a visit at Liverpool, I had a favourable opportunity of witnessing the exercise of an extraordinary faculty possessed by a blind young woman, named Margaret M'Evoy; and I have been induced, by the request of my friends, to send the results of my experiments for insertion in your Journal.

Without pretending to give a medical report of this singular case, which an abler pen is preparing for the public, I shall briefly premise that Miss M'Evoy is a native of Liverpool, and about 17 years of age. She became blind in the month of June, 1816, from a disorder in the head, which was supposed to be water on the brain, and was treated as such: she was partially relieved by a discharge from the ears and nostrils. She has since experienced two returns of the same disease, and each time has been relieved by a similar discharge of fluid. A portion of this fluid has, I believe, been analyzed by Dr. Bostock. She has remained completely blind from the time of the first attack. She first discovered by accident, about the middle of October, 1816, that she could read by touching the letters of a book.

Having blindfolded her in such a manner that I was certain not a ray of light could penetrate to her eyes, I made the following experiments, most of which had not been tried before. I copy the results from notes taken on the spot, and nearly in the order in which they were made:—

Exper. I.—I presented to her six differently coloured wafers fastened between two plates of common window glass. She accurately named the colour of each. She pointed out, unasked, the cracks and openings in the wafers. Being asked, while touching the surface of the glass above the red

 wafer, wafer, if the substance under might not be a piece of red cloth or paper, she answered, "No, I think it is a wafer."

Exper. II.—She described the colour and shape of triangular, square, and semicircular wafers, fastened in like manner between two plates of glass.

Exper. III.—To the seven prismatic colours, painted on a card, she gave the following names: scarlet, buff, yellow, green, light blue, dark blue or purple, lilac. As the orange paint was much faded, the term buff was correctly applied to it.

Exper. IV.—The solar spectrum being thrown by a prism, first on the back, and then on the palm, of her hand, she distinctly described the different colours, and the positions which they occupied, on her hands and fingers. She marked the moments when the colours became faint, and again vivid, by the occasional passage of a cloud. On one occasion she observed that there was something black upon her hand; but, perceiving it to move, she said it was the shadow of her own fingers, which was correct. The prismatic colours have afforded her the greatest pleasure which she has experienced since her blindness; the violet rays were the least pleasant. She never saw a prism in her life.

Exper. V.—The prism being put into her hands, she declared it was white glass; but, on turning it, she immediately said, "No, it is not; it is coloured; it has colours in it:" and she traced with her finger what she called "bent stripes of colours." She could discover no colours on that side of the prism on which the direct rays of light fell.

Exper. VI.—She perceived the coloured rings formed by pressing together two polished plates of glass. She said she felt them at the edge of her fingers flying before them.

Exper. VII.—Several attempts were made to ascertain whether she could discover colours in the dark, by presenting differently coloured objects to her hands, concealed under a pillow. She always failed; every thing appeared black. On one occasion she said a green card was yellow.

Exper. VIII.—She read a line or two of small print by feeling the letters. She next read through a convex lens at the distance of nine inches from the book. The principal focal length of the lens is 14 inches. While reading, she gently rubs the upper surface of the lens with the tips of her fingers; she reads much easier through the lens than without it; she says the letters appear larger, and as if they were printed on the glass. A pen-knife was laid on the line which she was reading, and she immediately perceived and named it.

Exper. IX.—A concave lens being put into her hands, she tried tried to read through it at the distance of seven or eight inches, but said that the letters were all confused. As she moved the lens gradually towards the book, she at length perceived the letters, but observed that they were very small. She could not read easily until the glass was laid on the paper.

Exper. X.—She read common print by feeling on the upper surface of a piece of common window glass held 12 inches from the book. At a greater distance she could not read; but could read much easier when the glass was brought nearer to the book. In like manner she perceived through the glass several coins spread out before her; told which had the head, which the reverse upwards; pointed out the position of the arms, crown, &c.; read the dates; and observed, unasked, that one half-guinea was crooked.

Exper. XI.—On applying her fingers to the window, she perceived two newly cut stones, of a yellow colour, lying one on the other, at the distance of 12 yards. She described a workman in the street, two children accidentally passing by, a cart loaded with barrels of American flour, another with loaves of sugar, a third empty, a girl with a small child in her arms, &c. One of the company being sent to place himself in different positions, she marked every change of position as soon as any one with his eye-sight could have done. A middle-sized man at the distance of 12 yards did not appear, she said, above two feet high. As he approached nearer, she observed that she felt him grow bigger. All objects appeared to her as if painted on the glass.

Exper. XII.—A stone ornament in the shape of an orange she took for a real orange, feeling through the plane glass at the distance of two or three inches; at the distance of 15 inches, it appeared no larger than a nut; at 30 inches distance, it was diminished to the size of a pea, the brightness of the colour remaining undiminished.

Exper. XIII.—On touching a plane glass mirror, she said that she felt the picture of her own fingers, and nothing else.

Exper. XIV.—Holding a plate of plane glass three or four inches before the mirror, she was then enabled to perceive the reflected image of herself. When the mirror was gradually removed further off, she said her face diminished. All objects constantly appear as a picture on the glass, which she touches.

Exper. XV.—She perceived through a plane glass, as before, the image of the sun reflected from a plane mirror; also the sun itself. She said that she was not dazzled with it, but found it very pleasant.

Exper. XVI.—She accurately described the features of two persons, whom she had never seen before, holding the plane plane glass at the distance of three or four inches from the face.

Exper. XVII.—Several small objects were held over her head. She perceived them all through her plane glass. On one occasion she asked, doubtingly, if a three-shilling piece was not a guinea? but, raising the glass, and bringing it nearer to the object, she corrected her error.

Exper. XVIII.—She was unable to distinguish colours by the tongue; but, holding between her lips the red, yellow, blue, and white petals of different flowers, she told the colour of each accurately.

Exper. XIX.—She accurately distinguished polished glass from natural crystals by the touch. She declared three several trinkets to be glass, which were believed to be stone: being tried by a file afterwards, they proved to be paste. She also distinguished between gold, silver, brass, and steel; likewise between ivory, tortoise-shell, and horn. "Gold and silver," she said, "feel finer than the other metals: crystals feel more solid, more firm, than glass."

Exper. XX.—She could not discover, by feeling, any difference between pure water and a solution of common salt in water.

These experiments were frequently repeated and varied, during the space of three days that I had the opportunity of seeing her, with the same results.

I must observe that this faculty of distinguishing colours and objects is more perfect at one time than at another: sometimes it suddenly and entirely fails; then every thing, she says, appears black. This sudden change seems like to what she remembers to have experienced when a candle has been extinguished, leaving her in the dark.

She says that she has not been taught by any one to distinguish colours by her fingers; but that, when she first perceived colours by this organ, she felt convinced that they were such and such colours, from the resemblance of the sensations to those which she had formerly experienced by means of the eye.

From the preceding facts, it appears evident that Miss M'Evoy has perceptions, through the medium of her fingers, similar to those which are usually acquired through the medium of the eye. With respect to the manner how she acquires them, and the necessity of an intermediate transparent substance when she does not actually touch the object, I shall offer no conjecture.

I have only further to add, that she has no apparent motive for attempting to impose upon those who visit her, were such an imposition practicable. She receives no remuneration from visitors. On the contrary, the mere presence of a stranger agitates her considerably for a time, so very weak and delicate is her state of health. Any noise or bustle affects her still more painfully: and I am ashamed to say that some of her visitors have showed a great and culpable disregard for her feelings, and subjected her to much unnecessary inconvenience.

*Stonyhurst; Aug.* 25, 1817.

---

*For the London Medical and Physical Journal.*

*On Wounds of the Stomach.*

